# Integrating Experimental Toxicology and Machine Learning to Model Levonorgestrel-Induced Oxidative Damage in Zebrafish

**DOI:** 10.3390/toxics13090764

**Published:** 2025-09-09

**Authors:** İlknur Meriç Turgut, Melek Yapıcı, Dilara Gerdan Koc

**Affiliations:** 1 Department of Fisheries and Aquaculture Engineering, Faculty of Agriculture, Ankara University, 06110 Ankara, Türkiye; melek_yapici@hotmail.com; 2 Department of Agricultural Machinery and Technologies Engineering, Faculty of Agriculture, Ankara University, 06110 Ankara, Türkiye; dgerdan@ankara.edu.tr

**Keywords:** synthetic progestins, ecotoxicity, dose–time response, redox biomarkers, ML-based toxicological modeling, supervised learning, risk assessment

## Abstract

Levonorgestrel (LNG), a synthetic progestin widely used in pharmaceuticals, is increasingly recognized as an emerging aquatic contaminant capable of exerting adverse biological effects beyond endocrine disruption. Acting in a xenobiotic-like manner, LNG may perturb redox homeostasis and induce oxidative stress in non-target species. To elucidate these mechanisms, this study integrates experimental toxicology with supervised machine learning to characterize tissue-specific and dose–time related oxidative responses in adult Zebrafish (*Danio rerio*). Fish were exposed to two environmentally relevant concentrations of LNG (0.312 µg/L; LNG-L and 6.24 µg/L; LNG-H) and a solvent control (LNG-C) for 24, 48, and 96 h in triplicate static bioassays. Redox biomarkers—superoxide dismutase (SOD), catalase (CAT), glutathione peroxidase (GPx), and malondialdehyde (MDA)—were quantified in liver and muscle tissues. LNG-H exposure elicited a time-dependent increase in SOD activity, variable CAT responses, and a marked elevation in hepatic GPx, with sustained MDA levels indicating persistent lipid peroxidation. Five classification algorithms (Logistic Regression, Multilayer Perceptron, Gradient-Boosted Trees, Decision Tree and Random Forest) were trained to discriminate exposure outcomes based on biomarker profiles; GBT yielded the highest performance (96.17% accuracy), identifying hepatic GPx as the most informative feature (AUC = 0.922). Regression modeling via Extreme Gradient Boosting (XGBoost) further corroborated the dose- and time-dependent predictability of GPx responses (R^2^ = 0.922, MAE = 0.019). These findings underscore hepatic GPx as a sentinel biomarker of LNG-induced oxidative stress and demonstrate the predictive utility of machinelearning-enhanced toxicological frameworks in detecting and modeling sublethal contaminant effects with high temporal resolution in aquatic systems.

## 1. Introduction

Endocrine-disrupting chemicals (EDCs) comprise diverse natural and synthetic compounds that capable of perturbing hormonal signaling and endocrine homeostasis across a wide spectrum of aquatic vertebrates and invertebrates [1,2,3,4,5]. Within this broad class, gestagens—a subclass of steroidal hormones including progestogens, progestins, and synthetic derivatives—have emerged as pollutants of particular toxicological concern due to their pharmaceutical origin [6], recalcitrance in aquatic matrices [7], and pronounced biological potency at environmentally relevant concentrations [8,9]. Although designed to selectively target progesterone receptors, many synthetic analogs display off-target interactions with androgenic and estrogenic receptors, thereby inducing pleiotropic endocrine disruption [10,11]. Given their extensive deployment in clinical and veterinary practice, gestagens are discharged into wastewater systems [12], where conventional treatment technologies exhibit limited efficacy in their removal [7]. This has led to these compounds being routinely detected in surface waters at concentrations spanning the low nanogram to microgram per liter range levels [13,14] sufficient to elicit sublethal severe biological effects in aquatic biota [6,15]. Notably, their ability to disrupt female reproduction at very low concentrations places progestins, the most concerning pharmaceutical class in aquatic systems after ethinylestradiol [16] and therefore, motivating our focus on LNG.

LNG—a 19-nortestosterone-derived synthetic progestin—has garnered particular scrutiny owing to its high receptor affinity, physicochemical stability, and environmental ubiquity [8,17]. Unlike endogenous steroids, LNG exhibits substantial resistance to abiotic and biotic degradation [9], facilitating its persistence and bioavailability in effluent-impacted ecosystems [18]. Upon entry into aquatic organisms, LNG has been shown to provoke a wide array of physiological and developmental disturbances [19], positioning it as a high-priority contaminant within the broader context of EDC risk assessment.

Exposure-based studies in both fish and amphibians have demonstrated that LNG and structurally related gestagens impair critical reproductive processes, including sexual differentiation [10,15,20], vitellogenin synthesis [21], and gonadal development [22]. The dose-dependent decreases in fecundity and fertility, reduced gonadosomatic index were also observed for Fathead minnow (*Pimephales promelas*) females [23]. Fuentes et al. [24] reported hyperactivity and aberrant neurogenesis at 5 ng/L in Zebrafish (*D. rerio*) larvae, while Teigeler et al. [25] noted an all-male sex ratio at 1.64 ng/L with reduced 11-keto testosterone and complete masculinization at 5.45 ng/L for adults. Moreover, LNG has been shown to profoundly disrupt the hypothalamic–pituitary–thyroid (HPT) axis in the African clawed frog (*Xenopus laevis*), leading to altered thyroid function and developmental anomalies—effects that extend beyond reproduction [15,26]. Complementary molecular evidence from teleosts reveals that such exposures dysregulate key regulatory genes within both the hypothalamic–pituitary–gonadal (HPG) and HPT axes [19,27], initiate gonadal anomalies and sex reversal [28,29], impair gametogenesis [30], diminish fecundity and mating behavior [31,32], and alter steroidogenic gene expression [33,34]. These mechanistic modulations are corroborated by the findings in both the African clawed frog (*X. laevis*) and Zebrafish models, in which LNG exposure has been shown to induce sex reversal and reduce mating success, effects frequently associated with sustained downregulation of reproductive gene networks [26,35]. An additional key point is that co-exposure with other gestagens can amplify reproductive impairments, indicating additive or even synergistic endocrine toxicity [36,37]. These multifaceted disruptions emphasize the ED- potential of synthetic gestagens and raise concerns for population-level consequences in aquatic ecosystems, particularly under chronic or mixture exposures [38,39].

Beyond established endocrine effects, mounting evidence suggests that their toxicological scope encompasses non-reproductive pathways, as pro-oxidant activity, perturbations of cellular redox balance and mitochondrial integrity [40,41,42,43]. In this context, LNG dysregulates redox-sensitive signaling cascades—primarily through the disruption of mitochondrial function [40] and the activation of endoplasmic reticulum stress responses [44] ultimately challenging cellular redox homeostasis and diminishing antioxidant defense capacity. As well, the oxidative effects of LNG tend to be tissue-specific and temporally dynamic; liver and gill tissues often exhibit heightened sensitivity due to their involvement in detoxification and environmental exchange processes [40,45]. While not a primary site of xenobiotic biotransformation, muscle acts as a sentinel of systemic oxidative stress under chronic exposure.

A comprehensive understanding of tissue-specific alterations must account for embryogenesis, a developmental stage marked by heightened susceptibility of endocrine and morphogenetic pathways. Compelling evidence indicates that LNG compromised Zebrafish embryogenesis at multiple levels of biological organization. Parental exposure reduces survival, growth, and normal development even in unexposed progeny [46], while acute to chronic toxicity has been associated with transcriptional dysregulation of genes involved in embryogenesis, immunity, lipid metabolism, and transport, contributing to early mortality within the first 72 h [47,48]. LNG exerts multi-level developmental toxicity, it modulates *pgr*, *ar*, *mr*, *gr*, and *hsd17ß3* above 2 ng/L, compromising brain and gonadal differentiation [49], and it induces *cyp19a1b* in radial glial cells, an estrogenic response with profound implications for neurogenesis and developmental patterning [33]. Binary mixtures with ethinylestradiol exacerbate these effects by disturbing the HPG axis and circadian signaling, producing delayed hatching, growth deficits, and malformations [37], while NGT ≥ 5 ng/L alters HPT axis transcripts in a concentration- and exposure-dependent manner [27]. Early-life exposure also perturbed locomotor and circadian networks, with reduced and altered *per1a* and *nr1d2a* expression at 16 ng/L via the PR/GR pathways, effects which are reversible with mifepristone [50]. Fuentes et al. [24] reported behavioral outcomes, with embryos exposed to 5 ng LNG exhibiting anxiety-like hyperactivity, heightened thigmotaxis, and elevated heart rates despite unaffected hatching.

The oxidative impairments of synthetic gestagens, as evidenced by elevated malondialdehyde (MDA) levels and compromised antioxidant enzyme activity in Nile tilapia (*Oreochromis niloticus*), goldfish (*Carassius auratus*), and Zebrafish [51,52,53]. Notably, sublethal LNG exposure in Zebrafish compromises mitochondrial respiration, augments reactive oxygen species (ROS) generation, and attenuates antioxidant capacity, hallmarks of a definitive redox-active mode of toxicity [40,41]. Comparable sensitivity has also been documented in the surf clam, *Mactra veneriformis*, with oxidative damage in digestive tissues emphasized the broader vulnerability of aquatic taxa to gestagen-induced stress [5]. A compelling study revealed that LNG bioaccumulation elevates hydrogen peroxide, stimulates antioxidant enzyme activity, and promotes lipid peroxidation in green microalga (*Chlorogonium elongatum*), highlighting primary producers’ susceptibility and potential ecosystem-wide ramifications from the food web base [54].

Machine learning (ML), a subset of artificial intelligence, has emerged as a transformative tool in environmental toxicology, offering data-driven approaches to predict toxicity with efficiency and mechanistic depth [55,56]. Widely used in pharmacology [57], materials science [58], biomedicine [59], and autonomous technologies [60], ML now enables high-throughput modeling of dose- and time-dependent effects from large-scale biochemical and genomic data [61,62], advancing aquatic toxicology by elucidating redox pathways, tissue-specific responses, and risk assessment in Zebrafish [63,64]. Nonetheless, challenges persist, including limited training data, underrepresentation of aquatic species, and restricted generalizability to novel compounds [65,66]. The promise of ML in aquatic toxicology is well recognized, as echoed by Wang et al. [67], whose analysis illuminates both advances in Zebrafish toxicity prediction and methodological gaps—particularly dataset scope and chemical diversity—that this study seeks to address.

Through ML approaches in forecasting toxicological outcomes, Zebrafish is the most popular candidate, due to their ability to model complex [68], multidimensional biological responses with high precision and predictive power. Macrae and Peterson [69] emphasized the utility of Zebrafish as a systems pharmacology model, paving the way for integrative computational strategies. Building on this foundation, Tal et al. [70] highlighted the translational value of Zebrafish-based ML models in toxicogenomic applications, while in parallel, Lin and Chou [71] also detailed the broader applicability of ML across toxicological disciplines, reinforcing its role in pollutant classification. Of particular note, Schwartz et al. [72] employed a suite of supervised ML models (RF, XGBoost, SVM and LR) to predict tissue-specific toxicity in Zebrafish embryos, achieving high accuracy and exemplifying ML’s integrative value in ecotoxicology.

With oxidative disturbances increasingly attributed to LNG, key mechanistic uncertainties remain in our understanding of its dose-dependent, time-resolved, and tissue-specific impacts in aquatic vertebrates. By coupling redox-sensitive biomarker analysis with supervised machine learning, this work intends to deepen mechanistic insight into LNG-induced redox disruption and to develop a robust predictive platform for ecotoxicological risk evaluation in freshwater realms as an inaugural application.

## 2. Materials and Methods

### 2.1. Empirical Toxicology Dataset

An empirical toxicology dataset was generated through static exposure trials implemented under regulated laboratory settings to ensure procedural consistency to evaluate the sublethal effects of LNG on adult Zebrafish. Fish were exposed to two environmentally relevant concentrations of LNG (0.312 and 6.24 µg/L) including a solvent-treated control group across three defined time points (24, 48, and 96 h) and data collection encompassed key biomarkers—including SOD, CAT, GPx, and MDA—sampled from liver and muscle tissues pertinent to oxidative stress and toxicological assessment. All experiments were performed in triplicate and followed OECD guidelines and institutional animal care protocols to ensure reproducibility, statistical robustness, and data integrity. This dataset subsequently served as the basis for supervised machine learning models designed to predict oxidative stress outcomes based on integrated biochemical parameters.

### 2.2. Test Chemical

The synthetic progestin LNG (C_21_H_28_O_2_; CAS No. 797-63-7; purity ≥ 99%), commercially recognized as d(-)-Norgestrel, was procured from Sigma-Aldrich (Steinheim, Germany). A primary stock solution was prepared by dissolving LNG in 0.1% (*v*/*v*) ethanol, employed as a carrier solvent. In alignment with environmentally relevant exposure levels previously documented by [73], two nominal concentrations were selected to represent low (0.312 µg/L) and high (6.24 µg/L) exposure scenarios. All working solutions were freshly diluted from the stock solution immediately prior to application to ensure dosing precision and chemical integrity throughout the experimental procedures.

### 2.3. Maintenance of Zebrafish and Exposure Procedure

Adult (1+ year-old) mixed-sex AB wild-type Zebrafish (*Danio rerio*, Hamilton, 1822; *n* = 540) were obtained from a local breeder (AKSU Akvaryum, Ankara, Türkiye), with a mean body weight of 7.24 ± 0.19 g and a mean total length of 3.02 ± 0.27 cm. All experimental procedures were conducted at the Department of Fisheries and Aquaculture Engineering, Ankara University (Ankara, Türkiye). Prior to chemical exposure, fish underwent a 15-day acclimation period in 96 L aerated glass aquaria under standardized laboratory conditions. For each treatment group, three replicate aquaria were randomly selected, thoroughly cleaned, and disinfected to ensure hygienic conditions and prevent cross-contamination. Municipal tap water, aged for 30 days to allow for complete chlorine dissipation, was used as the culture medium. Water quality parameters—including dissolved oxygen (DO_2_), temperature (°C), pH, oxidation-reduction potential (ORP), ammonia (NH_3_-N), nitrate (NO_3_-N), nitrite (NO_2_-N), hardness, and alkalinity—were measured prior to exposure in each group with three replicates and two parallel measurements, in accordance with APHA guidelines [74] (Table 1). Fish were fed twice daily to ad libitum with a commercial flake diet (Tetramin^®^ flakes, Tetra GmbH, Germany) during acclimation, but feeding was withheld 24 h prior to exposure to ensure gastrointestinal clearance and to avoid confounding metabolic influences on toxicokinetics and oxidative responses.

Experimental fish were exposed to 0.312 µg/L (LNG-L) and 6.24 µg/L (LNG-H) for three exposure durations: 24, 48, and 96 h [75]. Control groups (LNG-C) were maintained under identical conditions and received only the solvent (ethanol, 0.1% *v*/*v*), whereas experimental treatments involved direct exposure to LNG at the specified concentrations. Acute toxicity tests were performed using a static bioassay design with three replicates per treatment group. In accordance with standardized protocols for fish bioassays [76,77,78], each aquarium was stocked with 20 randomly selected fish of known body weight and length. All aquaria were securely covered with fine mesh netting to prevent escape and reduce environmental stress during handling and exposure.

### 2.4. Tissue Sampling and Homogenization Procedure

Sampling; upon each exposure period (24, 48, and 96 h), fish were euthanized via decapitation, in accordance with the American Veterinary Medical Association (AVMA) Guidelines for the Euthanasia of Animals and Directive 2010/63/EU. Immediately after euthanasia, liver and muscle tissues were dissected from both control and treatment groups and individually snap-frozen in liquid nitrogen and archived at −80 °C until subsequent quantification of oxidative stress biomarkers.

Homogenization; liver and muscle tissue specimens previously archived at −80 °C were allowed to equilibrate at ambient temperature (approximately 20 °C) for 5 to 15 min to facilitate controlled thawing. Precisely 0.1 g of each sample was weighed and transferred into 900 µL of potassium chloride (KCl) buffer (140 mmol/L). Tissue homogenization was performed on ice using a glass–Teflon homogenizer to ensure complete cellular disruption while preserving enzymatic integrity. The homogenates were centrifuged at 7000 rpm for 5 min at +4 °C and supernatants were carefully collected and aliquoted into sterile microcentrifuge tubes (50 µL per assay) and immediately stored on ice until subsequent enzymatic analyses. All procedures were performed under cold conditions to minimize oxidative artifact formation and to preserve the fidelity of oxidative stress biomarkers.

### 2.5. Antioxidative/Oxidative Stress Biomarkers

The activities of key antioxidant enzymes—SOD; (EC 1.15.1.1), CAT; (EC 1.11.1.6) and GPx; (EC 1.11.1.9)—were quantitatively assessed to evaluate oxidative stress responses. SOD activity was determined based on its ability to catalyze the dismutation of superoxide radicals into hydrogen peroxide and molecular oxygen. This assay utilized xanthine and xanthine oxidase to generate superoxide radicals, which subsequently reacted with 2-(4-iodophenyl)-3-(4-nitrophenol)-5-phenyltetrazolium chloride, forming a red formazan dye. The degree of inhibition of this reaction was used to quantify SOD activity [79]. CAT activity was evaluated by measuring the enzyme’s ability to catalyze the decomposition of hydrogen peroxide (H_2_O_2_). The reaction was halted by the addition of ammonium molybdate, which forms a yellow complex with the remaining H_2_O_2_ [80]. GPx activity was measured according to the method of Paglia and Valentine [81], which involves the GPx-catalyzed oxidation of reduced glutathione (GSH) by cumene hydroperoxide. The resulting oxidized glutathione (GSSG) was immediately converted back to GSH with the simultaneous oxidation of NADPH to NADP^+^. All enzymatic activities were determined spectrophotometrically; SOD at 560 nm (nitroblue tetrazolium salt reduction inhibition), CAT at 405 nm (H_2_O_2_-molybdate complex formation), and GPx at 340 nm (NADPH oxidation). The SOD and CAT activities were expressed in units (U) per mL and GPx activity in units (U) per L. One unit of SOD activity was defined as the amount of protein that inhibits the 50% of NBT salt reduction.

The MDA levels of liver and muscle homogenates were determined by using MDA-thiobarbituric acid (TBA) assay [82] which is used commonly for lipid peroxidation and values were expressed as nmol per L, the absorbance was measured at 532 nm.

### 2.6. Statictics

The observational data derived from the experimental trials were initially assessed for variance homogeneity, upon which the most appropriate analytical method was selected based on the distributional characteristics of the dataset. To facilitate intergroup (LNG-C, LNG-L and LNG-H) comparisons of distinct tissues (muscle and liver) across multiple exposure durations (24, 48, and 96 h), the non-parametric Kruskal–Wallis test was employed using SPSS Statistics software (version 26.0).

### 2.7. Data Pre-Processing

**Figure 1:**
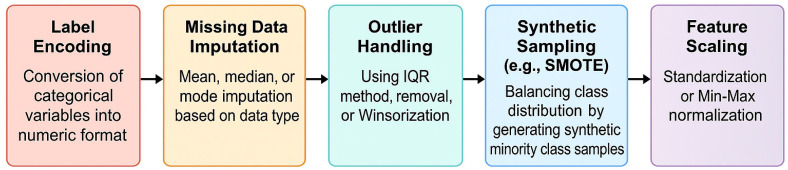
Flowchart of the data pre-processing pipeline including label encoding, imputation, outlier treatment, statistical analysis, feature scaling, and SMOTE-based resampling.

Prior to model development, the dataset underwent a comprehensive pre-processing workflow to ensure data quality, consistency, and suitability for ML analysis (Figure 1).

Categorical variables were first transformed into numerical format using label encoding (Figure 1, Step 1) to ensure compatibility with tree-based classifiers such as DT and RF, which are robust to the ordinal nature of encoded labels [83].

To address missing values, targeted imputation strategies were applied (Figure 1, Step 2): continuous variables were imputed using either the mean or median, depending on the symmetry of the distribution, while categorical variables were imputed using the mode. Outliers were identified using the interquartile range (IQR) method and were subsequently either excluded or adjusted through Winsorization (Figure 1, Step 3) to mitigate their influence on model training.

Descriptive statistics, including mean, standard deviation, and range, were computed to characterize the distribution of features. In addition, Pearson and Spearman correlation coefficients were calculated to evaluate linear and monotonic relationships among variables, respectively, and the Shapiro–Wilk test was used to assess normality (Figure 1, Step 4).

Feature scaling was then conducted using Min-Max normalization to rescale numerical attributes to a common range [0, 1], particularly to accommodate algorithms sensitive to differences in scale, such as MLP (Figure 1, Step 5).

Finally, the Synthetic Minority Over-sampling Technique (SMOTE) was applied to address class imbalance arising from uneven sample sizes among exposure groups (Figure 1, Step 6) [84]. SMOTE improves model generalizability by generating synthetic instances of the minority class through interpolation between existing samples and their nearest neighbors.

### 2.8. Machine Learning (ML) Models

A collection of supervised ML algorithms was employed to classify oxidative stress responses based on integrated biochemical markers. Each model was selected based on its unique capabilities in handling complex biological patterns.

#### 2.8.1. Logistic Regression (LR)

This algorithm is a fundamental statistical method that is employed to solve binary and multiclass classification problems. It is highly interpretable and computationally efficient due to the application of the logistic function, which models the probability of an instance belonging to a specific class. The robustness of logistic regression in managing linear relationships between independent and dependent variables has made it a widely used technique in a variety of fields, such as medical diagnosis and financial risk analysis [85]. It estimates the probability of a class using the sigmoid function:

$$P(y=1|x)=\frac{1}{1+e^{-\left(w^{T}x+b\right)}}$$

It assumes a linear relationship between the independent variables and the log-odds of the dependent variable [86].

#### 2.8.2. Multilayer Perceptron (MLP)

The MLP is a class of feedforward artificial neural networks capable of capturing complex, non-linear relationships within data through the use of one or more hidden layers. Each neuron in the network computes a weighted sum of its inputs and applies an activation function to introduce nonlinearity. A type of neural network capable of capturing complex relationships within the data by utilizing multiple layers and backpropagation techniques to optimize weights [87]. Mathematically, the output of a single neuron can be expressed as:

$$z=\phi \left(\sum_{i=1}^{n}\;\;w_{i}x_{i}+b\right)$$

where x_i_ represents the input features, w_i_ are the corresponding weights, b is the bias term, and ϕ(⋅) denotes the activation function. Common choices for ϕ include the sigmoid function $\phi \left(x\right)=\frac{1}{1+e^{-x}}$ the hyperbolic tangent, or the Rectified Linear Unit (ReLU), defined as ϕ(x)=max(0,x) The parameters w and b are optimized during training using the backpropagation algorithm in conjunction with gradient descent or its variants.

MLPs are particularly suited for modeling high-dimensional toxicological data, as they can effectively learn complex patterns across biochemical input features [88].

#### 2.8.3. Gradient-Boosted Trees (GBT)

An advanced ensemble learning technique that sequentially builds DT, each aimed at correcting the errors of its predecessor. This method excels in reducing bias and variance, leading to improved predictive accuracy [89]. GBT are based on the principle of boosting, an ensemble strategy that iteratively combines several weak learners—typically DT—to form a robust predictive model [90,91]. In contrast to bagging methods that assign equal importance to all data points through random sampling, boosting adaptively emphasizes samples that were previously misclassified, thereby directing the model’s learning capacity toward harder cases. Gradient boosting specifically builds DT in a stage-wise fashion, with each new tree trained to minimize the residual errors of the combined ensemble. This additive training process incrementally refines the predictive function by optimizing a predefined loss function, enhancing classification accuracy over successive iterations [92]. GBT is an ensemble method that sequentially adds DT to minimize the residual error of previous trees:

$$F_{m}(x)=F_{m-1}(x)+\gamma_{m}h_{m}(x)$$

It is known for high accuracy and flexibility in modeling non-linear relationships [89].

#### 2.8.4. Decision Tree (DT)

DT are hierarchical, rule-based models commonly employed in supervised learning for both classification and regression tasks. These models operate by recursively partitioning the dataset into subsets based on the values of input features. Each internal node represents a decision rule on a specific feature, while each leaf node corresponds to a final output label or value.

In classification problems, the splitting of nodes is typically guided by impurity measures such as Information Gain or the Gini Index, which evaluate the homogeneity of the resulting subsets [93]. The Gini Index quantifies the probability of incorrect classification of a randomly chosen element, and is defined as follows:

$$Gini\;Index=1-\sum_{i=1}^{c}{\left(P_{i}\right)}^{2}$$

Lower Gini values indicate purer nodes, thereby guiding the algorithm toward more informative splits.

#### 2.8.5. Random Forest (RF)

RF is an ensemble learning algorithm based on DT, designed for both classification and regression tasks. It operates by constructing multiple DT during training, each fitted on a different bootstrap sample of the dataset using the bagging (bootstrap aggregating) technique [94]. Unlike a single decision tree that may overfit to the training data, RF reduces variance and enhances generalization by aggregating predictions from diverse models trained on varied data subsets.

In classification tasks, each tree in the forest casts a vote for the predicted class, and the final decision is made by majority voting across all trees [95]. This can be formally expressed as follows:

$$\hat{y}=\arg\underset{c\in C}{max}\;\sum_{t=1}^{T}\;I\left(h_{t}\left(x\right)=c\right)$$

### 2.9. Model Performance Evaluation

**Figure 2:**
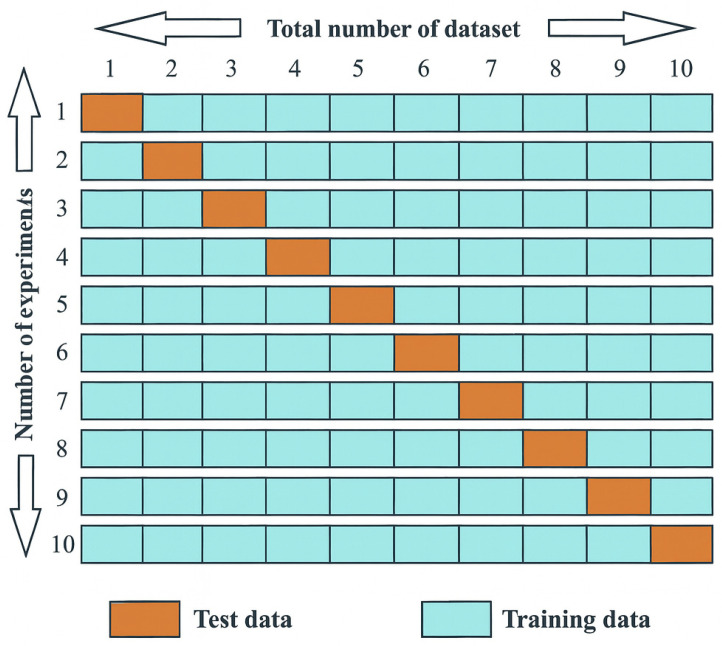
Schematic representation of the 10-fold cross-validation procedure used in model evaluation.

To comprehensively assess the performance of the classification models, multiple evaluation metrics were calculated, including accuracy, precision, recall (sensitivity), specificity, F1-score, Cohen’s Kappa, mean absolute error (MAE), root mean square error (RMSE), and area under the ROC curve (AUROC). Model evaluation was conducted using 10-fold cross-validation, where the dataset was divided into ten equal parts—nine parts were used for training and one for testing in each iteration (Figure 2). The final performance metrics were averaged across all folds to ensure robustness and minimize bias.

Accuracy indicates the overall proportion of correct predictions made by the following model:

$$Accuracy\;(1-Error)=\frac{TP}{\left(TP+TN+FP+FN\right)}$$

Precision, or positive predictive value (PPV), is the proportion of true positives among all predicted positives:

$$Precision=\frac{TP}{\left(TP+FP\right)}$$

Recall, also known as sensitivity or true positive rate (TPR), is the proportion of true positives among all actual positives:

$$Recall=\frac{TP}{\left(TP+FN\right)}$$

Specificity (true negative rate, TNR) measures the proportion of actual negatives correctly identified:

$$Specifity=\frac{TP}{\left(TN+FP\right)}$$

F1-score is the harmonic mean of precision and recall, providing a balanced metric in cases of class imbalance:

$$F-Measure=\frac{2\times Precision\times Recall}{Precision+Recall}$$

Cohen’s Kappa (κ) is a chance-corrected metric of classification agreement. It considers the likelihood of agreement occurring by chance:

$$(\kappa )=\frac{P_{0}-P_{e}}{1-P_{e}}$$

where $P_{0}$ is the observed agreement and $P_{e}$ is the expected agreement by random chance. Kappa values range from −1 (complete disagreement) to 1 (perfect agreement), with values above 0.6 generally considered substantial.

Mean Absolute Error (MAE) reflects the average magnitude of prediction errors without considering their direction:

$$MAE=\frac{1}{n}\sum_{i=1}^{n}\;\left|y_{i}-{\hat{y}}_{i}\right|$$

Root Mean Square Error (RMSE) is more sensitive to larger errors due to squaring:

$$RMSE=\sqrt{\frac{1}{n}\sum_{i=1}^{n}\;\;{\left(y_{i}-{\hat{y}}_{i}\right)}^{2}}$$

AUROC (Area Under the ROC Curve) evaluates the model’s discriminative power between classes by plotting the true positive rate (TPR) against the false positive rate (FPR):

$$ROC=\int_{0}^{1}\;\;\frac{\left(TP/TP+FN\right)}{\left(FP/FP+TN)(x\right)}dx$$

These performance indicators collectively provided a comprehensive view of classification effectiveness, reliability, and generalization across biochemical markers related to oxidative stress.

**Figure 3:**
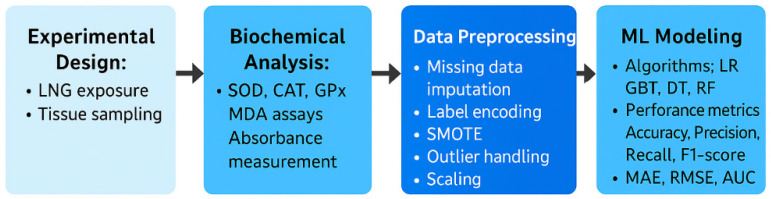
Overview of the experimental and computational workflow followed in the study.

To visually summarize the methodology employed in this study and enhance clarity regarding the analytical workflow, the diagram illustrates the key stages, including LNG exposure, biochemical analyses, data preprocessing, machine learning model development, and performance evaluation (Figure 3).

This integrative approach combines experimental toxicology and artificial intelligence techniques to predict oxidative stress responses induced by LNG.

## 3. Results

### 3.1. Empirical Outcomes for Antioxidant/Oxidant Biomarkers

The findings presented herein are derived from empirical toxicological analyses designed to elucidate the tissue-specific, dose-dependent, and temporal dynamics of antioxidant and oxidant responses following LNG exposure in adult Zebrafish. SOD demonstrated distinct activity patterns across tissues, exposure durations, and LNG concentrations. The highest level of SOD was 384.25 U/mL for 24 h LNG-C group in liver tissue, followed by a gradual decline under prolonged exposure and remarkably the lowest hepatic SOD activity (117.00 U/mL) occurred in the 96 h LNG-C group. However, SOD activity progressively increased over time under high-dose exposure (LNG-H), peaking at 96 h (381.55 U/mL) for muscle tissue. This trend suggests a time-dependent upregulation of SOD activity under high oxidative load, particularly in muscle, while the liver showed early antioxidant mobilization followed by depletion or suppression with extended exposure. CAT activity under LNG-L declined at 24 and 48 h for muscle, with partial recovery at 96 h and a reduction was also noted at 24 h under LNG-L in liver tissue, but enzyme activity was elevated at 48 and 96 h, indicating delayed hepatic CAT induction. For exposure to LNG-H, CAT activity declined consistently across all time points in both tissues, suggesting dose-dependent enzymatic suppression, possibly due to oxidative damage exceeding enzymatic capacity. GPx showed a robust dose–time response in muscle tissue, GPx activity was modest at early time points but surged to 515.83 U/L at 96 h under LNG-H. The response was even more pronounced, with GPx activity reaching 1055.81 U/L at 96 h in the LNG-H group for liver samples. These results indicate a strong dose- and time-dependent induction of GPx, particularly in hepatic tissue, likely reflecting compensatory activation in response to sustained oxidative insult. Lipid peroxidation, measured via MDA levels, further supported these trends at 24 and 48 h and MDA levels were generally low in both LNG-C and exposed groups (LNG-L and LNG-H). However, at 96 h, muscle MDA increased following to LNG-L exposure, while unexpectedly declining under LNG-H—possibly reflecting antioxidant activation or lipid damage repair. MDA levels of liver specimens, for LNG-L were lower than in muscle, while LNG-H exposure produced comparable MDA levels across tissues, suggesting convergence of oxidative damage under prolonged, high-intensity exposure. These results highlight a dose- and tissue-specific sensitivity to peroxidative damage, with liver tissue exhibiting higher oxidative responsiveness. The time-resolved, dose-dependent, and tissue-specific patterns observed under LNG exposure underscore the capacity of this gestagen to induce oxidative stress through differential modulation of enzymatic defenses.

### 3.2. ML Model Outcomes

The performance of individual ML models was assessed using a suite of classification metrics, including accuracy, precision, recall (sensitivity), specificity, F1-score (Table 2 and Table 3), and AUC-ROC. The application of the SMOTE effectively addressed class imbalance, enhancing the detection of minority class instances without compromising overall accuracy.

Categorical variables were processed via label encoding, preserving compatibility with tree-based algorithms. Missing values were imputed using distribution-aware strategies, maintaining data integrity and improving generalizability. Furthermore, feature scaling through normalization and standardization enabled efficient training convergence, particularly in algorithms sensitive to scale variations such as MLP.

Among all tested classifiers, the GBT model demonstrated the best performance with 96.17% accuracy and a Cohen’s Kappa of 0.923, indicating both high precision and class discrimination. RF closely followed with 94.97% accuracy and a kappa value of 0.899, while the DT model showed satisfactory results (93.47%, κ = 0.868).

In contrast, MLP and LR showed weaker performance, with LR displaying the lowest accuracy (82.06%) and kappa (0.642). These findings underscore the superior robustness and discriminative capacity of ensemble models, particularly GBT and RF, in modeling oxidative stress responses.

GBT achieved the best performance in both muscle and liver tissue classification, with recall, precision, and F1-scores exceeding 96%. The RF model also yielded strong results, particularly in liver classification (F1 = 0.951), positioning it as a reliable alternative. In contrast, DT provided acceptable but slightly lower outcomes, while MLP and LR showed notably reduced predictive power—especially in muscle classification—with lower F1-scores.

Table 4 presents the performance metrics of the regression models constructed for liver tissue in relation to LNG exposure, taking into account both dose and duration. For each oxidative stress biomarker, the coefficient of determination (R^2^), MAE, and RMSE were calculated to evaluate model accuracy and explanatory capacity. GPx and MDA exhibited the highest R^2^ values, indicating strong responsiveness to the exposure variables. Remarkably, GPx also demonstrated the lowest MAE and RMSE values, suggesting superior predictive performance and minimal deviation from observed data. These findings highlight GPx as the most reliable and sensitive hepatic biomarker under the tested conditions, providing both robust statistical significance and biological relevance in modeling LNG-induced oxidative stress.

Regression analysis with XGBoost revealed that both LNG concentration and exposure time significantly influenced oxidative stress biomarkers in Zebrafish liver. GPx emerged as the most responsive and reliable biomarker (R^2^ = 0.922, lowest MAE and RMSE), followed by MDA. SOD showed moderate explanatory power (R^2^ = 0.810), indicating its role in early-phase antioxidant defense. CAT, while still statistically significant, exhibited lower predictability, potentially due to more complex temporal regulation. These findings suggest a dynamic and biomarker-specific oxidative stress response to LNG exposure.

In contrast to the regression-based findings, the classification analysis using ROC curves revealed that only GPx exhibited statistically significant discriminative power among the evaluated biomarkers. AUC values indicated that this significance was exclusive to liver tissue, confirming both the dose- and time-dependent responsiveness of GPx as well as its diagnostic value.

While MDA displayed strong predictive capacity under regression modeling, it did not achieve statistical relevance in classification metrics. This discrepancy suggests that MDA is more indicative of chronic biochemical alterations rather than acute exposure-based class separation.

**Figure 4:**
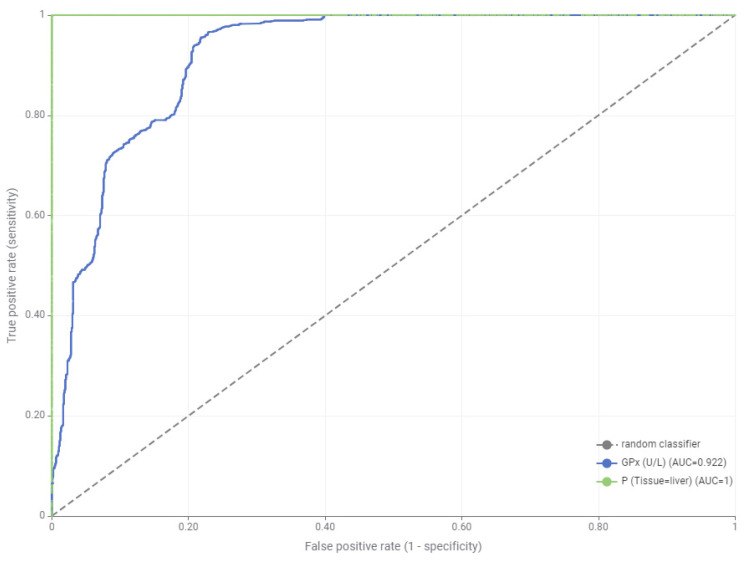
ROC analysis for liver tissue (GPx: Glutathione peroxidase; AUC: Area under the curve).

As shown in Figure 4, the ROC curve of GPx demonstrates a markedly higher AUC compared to other biomarkers, underlining its diagnostic superiority in distinguishing exposure groups. The remaining biomarkers failed to yield statistically significant AUC values, further emphasizing GPx as the most reliable and tissue-specific indicator of LNG-induced oxidative stress in Zebrafish liver.

ROC analysis revealed that GPx had the highest discriminative power (AUC = 0.922) in liver tissue, confirming its role as a sensitive and specific biomarker for LNG-induced oxidative damage.

### 3.3. Distributional Analysis of Oxidative Stress Biomarkers for Tissue and Dose Specifity

**Figure 5:**
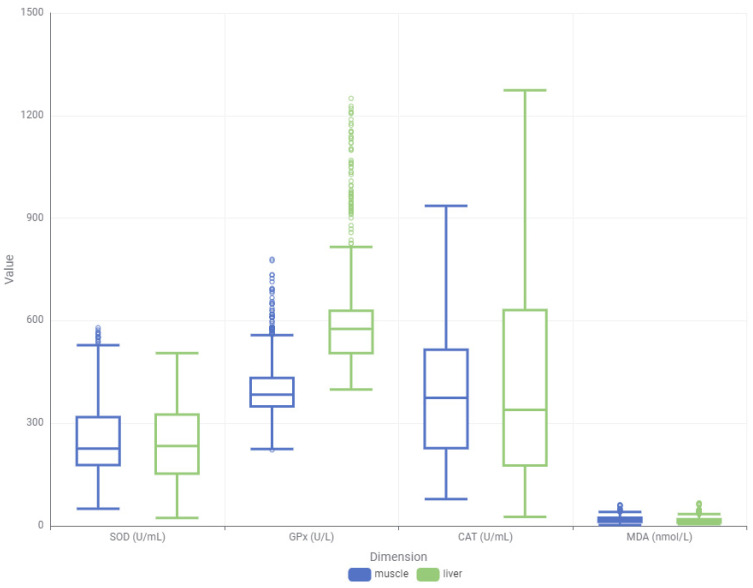
Box plot of oxidative stress biomarkers for muscle and liver tissues (SOD: Superoxide dismutase; GPx: Glutathione peroxidase; CAT: Catalase; MDA: Malondialdehyde).

In order to better understand the differential response patterns of oxidative stress biomarkers, both distributional (box plot) and density-based visualizations were employed across muscle and liver tissues (Figure 5). These plots revealed clear distinctions in the baseline values, distribution shapes, and variability of SOD, CAT, GPx, and MDA levels, suggesting tissue-specific response mechanisms to LNG exposure.

The box plot analysis illustrated that liver tissues generally exhibited a broader range and higher variance for most biomarkers, particularly GPx and CAT, when compared to muscle tissues. GPx levels in the liver were markedly elevated with several extreme values, indicating a strong and variable antioxidant response. CAT also showed extensive variability in the liver, consistent with its irregular temporal behavior.

**Figure 6:**
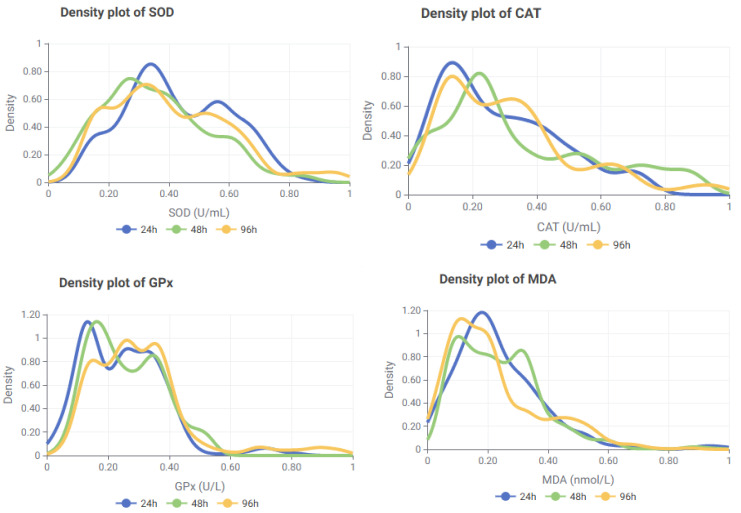
Density plot of SOD, CAT, GPx and MDA for time courses (SOD: Superoxide dismutase; GPx: Glutathione peroxidase; CAT: Catalase; MDA: Malondialdehyde).

To further investigate the kinetic patterns of biomarker expression, density plots were generated for each marker using the full dataset (n = 1164). These plots revealed biomarker-specific trends and modality differences that could not be fully captured by summary statistics alone (Figure 6).

The time-based density analysis revealed that each biomarker exhibited distinct response kinetics across the 24 h, 48 h, and 96 h exposure intervals. SOD showed a sharp early peak at 24 h, followed by a decline at 48 h and partial recovery at 96 h. This pattern, together with the bimodal distribution observed at all time points, supports its role as a rapid oxidative stress responder subject to phase-dependent regulation during prolonged exposure.

CAT displayed a two-phase activation profile. An early increase at 24 h was followed by a modest redistribution at 48 h and relatively stable activity at 96 h. These fluctuations suggest that CAT activity involves both immediate and sustained components, likely modulated by context-dependent activation and post-translational control mechanisms.

GPx exhibited the most prominent delayed-phase response. Activity was comparatively lower at 24 h but increased progressively at 48 h and 96 h, with broader distributions indicative of extended antioxidant engagement during later exposure stages. This pattern reinforces its role as a key defense enzyme in the late phase of oxidative stress.

MDA showed marked early-phase elevations at 24 h and 48 h, followed by a moderate decline and broader distribution at 96 h. This trend suggests strong lipid peroxidation during the initial exposure stages, with partial attenuation over time, consistent with its role as an indicator of both acute and sustained oxidative damage.

**Figure 7:**
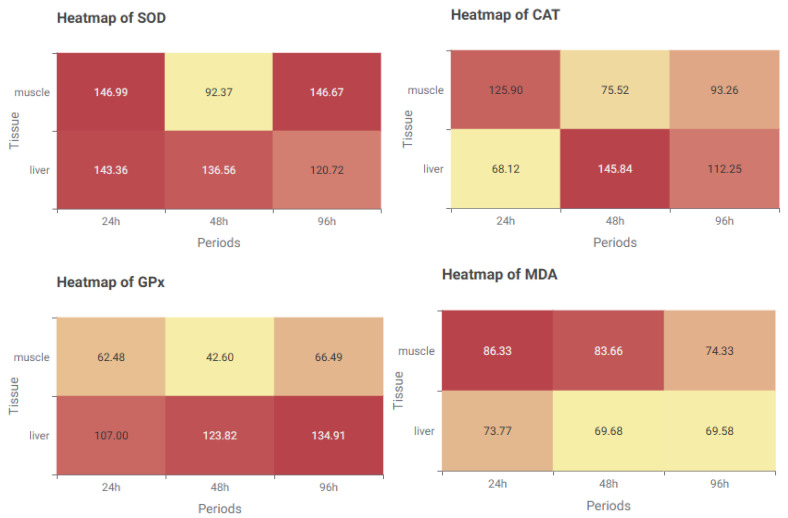
Heatmaps illustrating the average levels of oxidative stress biomarkers (SOD: Superoxide dismutase; GPx: Glutathione peroxidase; CAT: Catalase; MDA: Malondialdehyde) in liver and muscle tissues over three exposure periods. Color intensity reflects relative biomarker levels (lighter shades = lower values; darker shades = higher values).

Taken together, these time-based profiles indicate that SOD functions as an early-phase oxidative stress sentinel, CAT demonstrates dual-phase activation, GPx predominates during late-phase antioxidant defense, and MDA reflects early and persistent lipid peroxidation. These distinct patterns underscore the importance of integrating time-course resolution into biomarker-based toxicological modeling. To further illustrate these differences, a series of heatmaps was generated (Figure 7).

Distinct patterns emerged across biomarkers and GPx exhibited progressively increasing levels in liver tissue. MDA remained higher in muscle than in liver, with values decreasing over time in muscle and remaining relatively stable in liver. SOD showed high early-phase activity, particularly in muscle tissue, followed by a decrease at 48 h and partial recovery at 96 h. CAT presented a heterogeneous pattern, with muscle peaking early and liver peaking at 48 h. These visual trends support earlier statistical findings.

**Figure 8:**
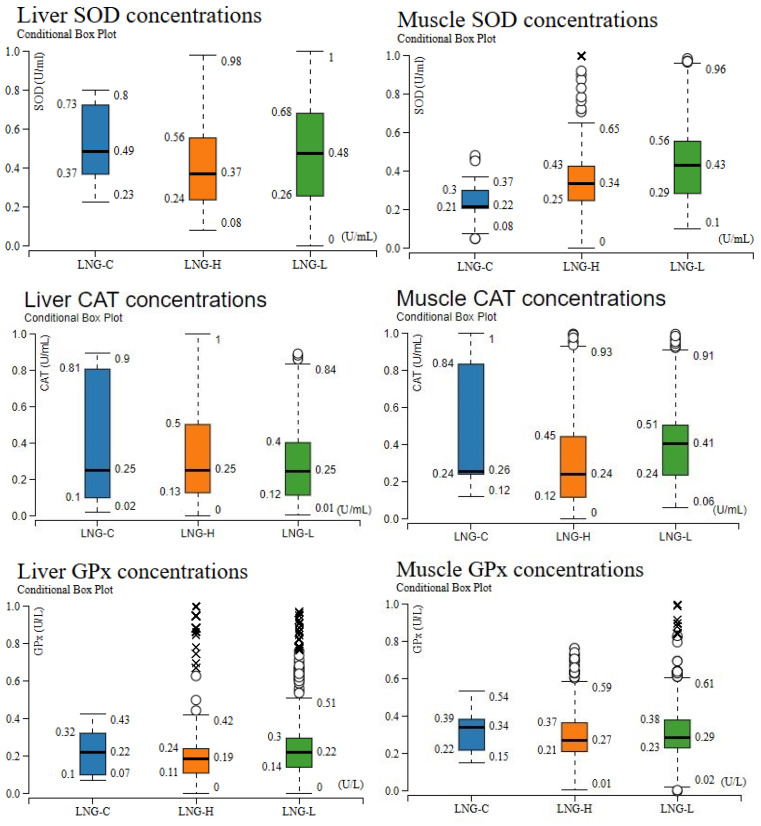

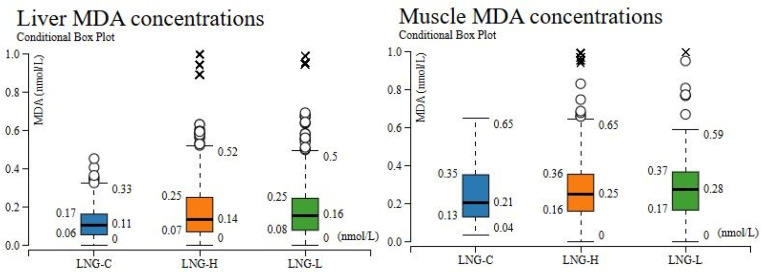
Variation in oxidative stress biomarkers (SOD: Superoxide dismutase; GPx: Glutathione peroxidase; CAT: Catalase; MDA: Malondialdehyde) for liver and muscle tissues following dose-dependent (LNG-C, LNG-H and LNG-L) exposure. Circles represent mild outliers (values outside 1.5× IQR), while crosses indicate extreme outliers (values outside 3× IQR).

The concentration-dependent modulation of oxidative stress biomarkers is depicted in Figure 8, summarizing SOD, CAT, GPx, and MDA distributions in liver and muscle tissues under control (LNG-C), low-dose (LNG-L), and high-dose (LNG-H) exposure. Across both tissues, LNG-C exhibited higher median antioxidant enzyme activities and lower MDA levels, whereas LNG-treated groups displayed enzymatic suppression and elevated lipid peroxidation, consistent with dose-dependent oxidative impairment.

SOD activity peaked in LNG-C but declined markedly in both LNG-H and LNG-L for the liver, while muscle SOD displayed greater variability under LNG-H, with slightly elevated medians in both exposure groups relative to control—suggesting tissue-specific SOD modulation. CAT activity displayed a consistent decline across both tissues in the treatment groups compared to controls and median CAT levels were highest in LNG-C, whereas LNG-H and LNG-L groups showed pronounced reductions for liver samples. In muscle tissue, CAT activity was similarly reduced in LNG-H, with partial recovery in LNG-L, indicating a possible adaptive response at lower exposure levels. For GPx activity, both liver and muscle tissues showed the highest median values in LNG-C. Exposure to LNG-H and LNG-L resulted in a significant reduction in GPx activity, with the high-dose group exhibiting the greatest variability, reflecting inter-individual differences in enzymatic antioxidant capacity under chemical stress.

MDA concentrations were minimal for LNG-C groups, increased in both LNG-H and LNG-L, reflecting enhanced lipid peroxidation. Both exposure groups showed increased MDA levels, with numerous outliers indicating pronounced oxidative damage in certain individuals. Notably, liver MDA values in LNG-H demonstrated a broader distribution compared to LNG-L, whereas muscle MDA increases were comparable between the two exposure groups.

## 4. Discussion

Based on the present data, LNG exposure unveiled profound interspecies divergence in oxidative stress enzyme dynamics, underlining species-specific antioxidant strategies and variable capacities to withstand xenobiotic-induced redox perturbations. High-dose LNG (6.24 µg/L) progressively elevated SOD activity in both liver and muscle, reflecting compensatory antioxidant upregulation under sustained oxidative stress for Zebrafish and Zebra mussel (*Dreissena polymorpha*) [73] contrasting with the grooved carpet clam (*Ruditapes decussatus*), where SOD remained unaffected despite clear stress indicators [41]. This divergence may relate to LNG’s physicochemical stability and high receptor affinity, which enable prolonged bioactivity and cumulative oxidative pressure in vertebrate tissues [8,9]. CAT responses were highly variable, mirroring patterns reported for other gestagens and species; marine medaka (*Oryzias melastigma*) exhibited NET-induced increases [40], whereas NGT (0.10–1000 ng/L) and combined LNG/NET exposures in the surf clam (*M. veneriformis*) and Zebrafish suppressed CAT [5,45,96]. Such bidirectional trends are consistent with the notion that mitochondrial and endoplasmic reticulum stress responses can either induce or inhibit antioxidant enzymes depending on oxidative load severity [42,44].

GPx was strongly induced under LNG-H—particularly in the liver—aligning with NET-driven increases in marine medaka (*O. melastigma*) [40] and reinforcing the liver’s role as a primary oxidative stress target due to its detoxification capacity [40,45]. Nonetheless, reductions in GPx have been documented in Zebrafish and the surf clam (*M. veneriformis*) exposed to other progestogens [5,96].

MDA levels displayed greater hepatic sensitivity here, paralleling NET-induced increases in marine medaka and elevated TBARs in the grooved carpet clam (*R. decussatus*) under LNG treatments [41]. These oxidative impairments align with broader evidence that enzyme-specific and species-dependent trajectories not only reflect divergent antioxidant strategies but also reveal evolutionary variability in redox resilience across vertebrates and invertebrates, thereby heightening susceptibility to additional environmental stressors such as hypoxia or temperature shifts [97]. In this context, interspecies divergence in oxidative stress responses cannot be attributed solely to exposure concentration but rather emerges from fundamental differences in uptake dynamics and intrinsic factors. In fish, disproportionately high LNG uptake may be facilitated by sex-steroid binding globulins in gill tissues, acting as molecular traps for circulating steroids and amplifying intracellular oxidative pressure. This precipitates a characteristic enzymatic profile marked by pronounced SOD induction, variable CAT modulation, and robust GPx activation. By contrast, uptake mechanisms in bivalves remain insufficiently resolved [73] and the absence of evidence for analogous binding proteins suggests a more constrained internal burden, congruent with the limited enzymatic adjustments described. Such uncertainty accentuates the imperative for continued investigation, situating such interspecies variability within an adverse outcome pathway (AOP) framework offers a mechanistic continuum linking molecular perturbations to population-level consequences, thereby enhancing their interpretive value for ecotoxicological risk assessment and predictive environmental modeling.

These biomarker trends provided the foundation for subsequent ML modeling, enabling predictive classification of LNG exposure scenarios and elucidation of tissue-specific oxidative trajectories with high temporal resolution. By coupling these experimental data with supervised ML algorithms, hepatic GPx emerged as the most sensitive and diagnostically informative biomarker. Among the algorithms tested, GBT achieved the highest classification accuracy (96.17%), and liver GPx yielded the highest ROC-AUC (0.922), outperforming muscle tissue markers. Furthermore, XGBoost regression modeling revealed a strong dose- and time-dependent relationship for liver GPx (R^2^ = 0.922, MAE = 0.019), confirming its role as a sentinel indicator of oxidative stress.

These results are particularly significant in light of prior research that has characterized endocrine and enzymatic disruptions following LNG exposure—such as altered CAT expression—but has largely overlooked tissue-specific oxidative stress biomarkers and computational modeling approaches [28,96]. Prominently, our study also found that muscle tissue biomarkers exhibited lower predictive power and temporal stability, suggesting that hepatic profiling offers a more robust and reliable window into xenobiotic-induced oxidative stress in aquatic vertebrates.

Comparable findings have been reported by Wang et al. [98], who used RF and XGBoost algorithms to model nanoparticle-induced oxidative damage in mussel tissues. Their study also identified GPx and MDA as highly predictive markers, with GPx demonstrating the strongest correlation with exposure intensity. These interspecies parallels further support the broader utility of GPx as a conserved and sensitive oxidative biomarker.

More broadly, ML applications in Zebrafish toxicology are increasingly being adopted to evaluate chemical-induced outcomes across a range of endpoints. For example, Wang et al. [67] emphasized the use of ML in predicting neurotoxicity and genotoxicity, while Tal et al. [70] employed quantitative structure–activity relationship (QSAR) models to explore acute toxicity and mode-of-action pathways. Schwartz et al. [72] reported that RF achieved 74% cross-validation accuracy when modeling transcriptomic responses associated with pancreatic toxicity in Zebrafish embryos—highlighting the organ-specific predictive capacity of ML.

These trends are echoed in comprehensive reviews by Macrae and Peterson [69], who underscored the relevance of automated phenotyping and deep learning in systemic toxicity detection, and Lin and Chou [71], who called for the integration of ML with physiologically based pharmacokinetic models and toxicogenomic data pipelines to enhance toxicological insight.

In parallel, multiple studies have demonstrated the versatility of ML for detecting and quantifying oxidative stress in Zebrafish. As a case in point, unsupervised clustering methods like UPGMA have been used to analyze Zebrafish locomotor patterns [99], and Google’s AutoML platform (version 1.0.0) has achieved high accuracy in detecting morphological abnormalities in fluorescent-labelled Zebrafish [100]. Fluorescent ROS probes [101] and genetically encoded sensors [102] have enabled real-time, in vivo imaging of oxidative processes. Additionally, ML models have successfully classified abiotic and biotic stressors [103], staged embryonic development automatically [104], and scored ototoxin-induced damage in Zebrafish hair cells [105]. These diverse approaches collectively enhance the applicability of Zebrafish as a high-throughput, redox-sensitive model for studying oxidative stress and its pathological consequences [106].

To further contextualize our findings, we compiled recent literature exploring both the biological and computational responses to LNG and similar endocrine-disrupting chemicals in Zebrafish (Table 5).

**Table 5:** Recent studies on LNG Toxicity and ML-Based Prediction for Zebrafish.

Study	Focus	Exposure Details	Key Findings	Statistical Significance/Model Accuracy
[40]	Morphological abnormalities	Exposure up to 120 hpf *	8 abnormal phenotypes and 8 organ features classified	mAP > 0.93; Accuracy > 0.86
[72]	Pancreatic toxicity and gene expression	Not specified	Chemical clusters identified affecting pancreatic pathways	RF accuracy 74%
[107]	Neuronal development (neuroendogenesis)	LNG: 5 ng; Estradiol: 100 ng; 5 days	↑ alpha-HUC+ neurons in hypothalamus and related regions	*p* < 0.001 (hypothalamus), *p* < 0.01 (preoptic area)
[108]	Acute toxicity prediction (QSAR/q-RASAR)	LC_50_: 0.790 mg/L (exp); 0.763 mg/L (pred)	Phenolphthalein identified as highly toxic	R^2^ = 0.886, Q^2^ = 0.814

* Abbreviations: hpf; hours post fertilization, mAP; mean Average Precision, QSAR; Quantitative Structure–Activity Relationship, q-RASAR; quantitative Read-Across Structure–Activity Relationship, LC_50_; lethal concentration for 50% of exposed organisms, R^2^; Coefficient of determination; Q^2^; Predictive squared correlation coefficient.

Our study expands upon this foundation by taking the advances of the current understanding of progestin-induced toxicity by establishing a validated ML framework for modeling oxidative biomarker dynamics and confirms the diagnostic value of liver GPx, underscore the superiority of hepatic versus muscular biomarkers in predictive performance, and emphasize the importance of temporally resolved, tissue-specific strategies in aquatic toxicology.

A further dimension requiring emphasis is, although, defining safe usage thresholds is critical for contextualizing the toxicity of LNG in aquatic ecosystems, a critical point emerging from the current body of knowledge is that LNG can exhibit deleterious impacts at extremely low concentrations with risk quotients often exceeding unity in surface waters. As summarized in Oropesa and Guimarães [48], a preliminary Environmental Risk Assessment (ERA) of LNG following the European Medicines Agency, EMEA [109] guidelines highlighted the significant environmental concern. Using measured environmental concentrations (MECs) of up to 38 ng/L in surface waters and 11 ng/L in groundwater, risk quotients (RQs) exceeded unity for surface waters, indicating a potential ecological risk. Critically, the lowest available effect concentration (EC10 = 0.28 ng/L) [110] falls well within environmentally observed levels and the calculated predicted no-effect concentration, PNEC for surface waters (0.000028 µg/L with an assessment factor of 10, or 0.0000056 µg/L with an assessment factor of 50) is substantially lower than measured MECs, reinforcing that LNG can impair physiological integrity of aquatic species at levels congruent with those measured in surface waters. Such findings support the argument that putative safety thresholds for LNG remain stringently low and defined by highly precautionary criteria and still uncertain without a stronger mechanistic basis. Notwithstanding that this study did not establish permissible exposure thresholds, it demonstrates that even low µg/L concentrations, orders of magnitude higher than environmental detections but within the range used in toxicological testing, provoke measurable redox disturbances in fish. Such mechanistic data are crucial to support ERA under the AOP framework and is increasingly recommended for assessing endocrine-disrupting compounds [111,112].

## 5. Conclusions

The inherent complexity and nonlinearity of tissue-specific trajectories highlight the necessity of predictive modeling to disentangle dose- and organ-dependent patterns. Integrating empirical biochemical profiling with such modeling not only facilitates the early detection of progestin-driven redox perturbations—often undetectable by conventional toxicological endpoints—but also enhances prediction accuracy and elucidates the variable contributions within classification frameworks. This methodological synergy underscores the value of advanced machine learning in data-rich biological contexts. Looking ahead, the incorporation of transcriptomic or proteomic datasets could further refine biomarker specificity, enhance cross-species applicability, and bolster environmental monitoring strategies for endocrine-active contaminants in freshwater ecosystems.

## Figures and Tables

**Table 1:** Water quality parameters across the treatments were presented as mean ± standard deviation (*n* = 6).

Treatment *	Time (h)	Parameter								
		DO_2_ (mg/L)	°C	pH	ORP(mV)	NH_3_-N (mg/L)	NO_3_-N (mg/L)	NO_2_-N (mg/L)	Hardness (mg/L)	Alkalinity (mg/L CaCO_3_)
LNG-C	24	7.15 ± 0.27	24.24 ± 0.54	8.37 ± 0.10	17.88 ± 2.47	0.16 ± 0.02	1.22 ± 0.36	0.19 ± 0.11	11.27 ± 2.46	7.00 ± 1.60
	48	7.24 ± 0.49	24.56 ± 0.51	8.32 ± 0.09	18.22 ± 2.15	0.18 ± 0.00	0.99 ± 0.47	0.06 ± 0.01	18.00 ± 1.70	4.00 ± 0.00
	96	7.13 ± 0.17	24.17 ± 0.16	8.38 ± 0.03	17.91 ± 1.88	0.15 ± 0.01	1.42 ± 0.32	0.16 ± 0.24	11.67 ± 1.87	6.00 ± 0.00
LNG-L	24	7.31 ± 0.29	24.13 ± 0.90	8.33 ± 0.04	15.68 ± 0.71	0.17 ± 0.01	1.07 ± 0.13	0.06 ± 0.00	17.67 ± 1.87	4.00 ± 0.00
	48	7.18 ± 0.43	24.71 ± 0.69	8.27 ± 0.05	15.92 ± 1.86	0.17 ± 0.00	1.04 ± 0.19	0.06 ± 0.00	18.00 ± 1.71	4.00 ± 0.00
	96	7.76 ± 0.43	24.78 ± 0.08	8.19 ± 0.09	14.61 ± 1.43	0.16 ± 0.03	1.46 ± 0.83	0.04 ± 0.01	9.33 ± 6.68	3.47 ± 0.82
LNG-H	24	8.33 ± 0.25	23.98 ± 0.37	8.09 ± 0.25	16.03 ± 7.49	0.09 ± 0.06	1.53 ± 0.53	0.08 ± 0.10	17.67 ± 0.78	4.00 ± 0.00
	48	8.29 ± 0.59	24.99 ± 1.33	8.15 ± 0.10	16.74 ± 3.57	0.12 ± 0.06	1.35 ± 0.51	0.05 ± 0.00	16.00 ± 1.21	4.00 ± 0.00
	96	7.87 ± 0.54	24.74 ± 0.64	8.16 ± 0.04	12.64 ± 1.43	0.14 ± 0.02	1.63 ± 0.43	0.03 ± 0.00	5.33 ± 1.97	3.67 ± 0.78

* Treatment: LNG-C; Control (ethanol, 0.1%), LNG-L; (0.312 µg/L), LNG-H (6.24 µg/L).

**Table 2:** The models’ overall classification performance.

Algorithm *	Accuracy (%)	Error (%)	Cohen’s Kappa
GBT	96.17	3.83	0.923
RF	94.97	5.03	0.899
DT	93.47	6.58	0.868
MLP	85.24	14.77	0.704
LR	82.06	17.96	0.642

* Algorithm: GBT; Gradient-Boosted Trees, RF; Random Forest, DT; Decision Tree, MLP; Multi-Layer Perceptron, LR; Logistic Regression.

**Table 3:** Class-wise performance evaluation of muscle and liver.

Model *	Class	Recall	Precision	Sensitivity	Specificity	F-Measure
GBT	Muscle	0.958	0.964	0.958	0.965	0.961
GBT	Liver	0.965	0.96	0.965	0.958	0.962
RF	Muscle	0.938	0.959	0.938	0.961	0.948
RF	Liver	0.961	0.941	0.961	0.938	0.951
DT	Muscle	0.937	0.928	0.937	0.931	0.932
DT	Liver	0.931	0.94	0.931	0.937	0.936
MLP	Muscle	0.833	0.858	0.833	0.87	0.845
MLP	Liver	0.847	0.847	0.87	0.833	0.859
LR	Muscle	0.856	0.791	0.856	0.787	0.822
LR	Liver	0.787	0.853	0.787	0.856	0.819

* Model: GBT; Gradient-Boosted Trees, RF; Random Forest, DT; Decision Tree, MLP; Multi-Layer Perceptron, LR; Logistic Regression.

**Table 4:** Evaluation of the effect of concentration and time on biomarkers with regression model.

Biomarker *	R^2^ **	MAE	RMSE
GPx	0.922	0.019	0.041
MDA	0.849	0.113	0.273
SOD	0.81	0.123	0.307
CAT	0.78	0.159	0.330

* Biomarker: GPx; Glutathione peroxidase, MDA; Malondialdehyde, SOD; Superoxide dismutase, CAT; Catalase. ** Abbreviations: R^2^; Coefficient of determination, MAE; Mean absolute error, RMSE; Root mean squared error.

## Data Availability

The datasets analyzed and/or generated during the current study are available from the corresponding author on reasonable request.

## Abbreviations

The following abbreviations are used in this manuscript:

EDCs	Endocrine disrupting chemicals
LNG	Levonorgestrel
LNG-C	Levonorgestrel-control
LNG-H	Levonorgestrel-high concentration
LNG-L	Levonorgestrel-low concentration
HPT	Hypothalamic–pituitary–thyroid
HPG	Hypothalamic–pituitary–gonadal
ROS	Reactive oxygen species
SOD	Superoxide dismutase
CAT	Catalase
GPx	Glutathione peroxidase
MDA	Malondialdehyde
ML	Machine learning
LR	Logistic regression
MLP	Multilayer perceptron
GBT	Gradient-boosted trees
DT	Decision tree
RF	Random forest
XGBoost	Extreme gradient boosting
SMOTE	Synthetic minority over-sampling technique
QSAR	Quantitative structure–activity relationship
q-RASAR	quantitative read-across structure–activity relationship
OECD	Organisation for Economic Co-operation and Development
SVM	Support vector machine
DO_2_	Dissolved oxygen
°C	Temperature
ORP	Oxidation-reduction potential
NH_3_-N	Ammonia
NO_3_-N	Nitrate
NO_2_-N	Nitrite
APHA	American Public Health Association
TSE	Türk Standartları Enstitüsü
AVMA	American Veterinary Medical Association
EU	European Union
KCl	Potassium chloride
H_2_O_2_	Hydrogen peroxide
GSSG	Oxidized glutathione
GSH	Reduced glutathione
NADPH	Nicotinamide adenine dinucleotide phosphate (reduced form)
NADP+	Nicotinamide adenine dinucleotide phosphate (oxidized form)
NBT	Nitroblue tetrazolium
IU	International units
U	Units
TBA	Thiobarbituric acid
IQR	Interquartile range
ReLU	Rectified linear unit
MAE	Mean absolute error
RMSE	Root mean square error
ROC	Receiver operating characteristic
AUROC	Area under the receiver operating characteristic curve
PPV	Positive predictive value
TPR	True positive rate
TNR	True negative rate
κ	Cohen’s Kappa
FPR	False positive Rate
R^2^	The coefficient of determination
UPGMA	Unweighted pair group method with arithmetic mean
AOP	Adverse outcome pathway
ERA	Environmental risk assessment
EMEA	European medicines agency
MECs	Measured environmental concentrations
RQs	Risk quotients
PNEC	Predicted no-effect concentration
